# A meta-analysis of the effect of a dexamethasone intravitreal implant versus intravitreal anti-vascular endothelial growth factor treatment for diabetic macular edema

**DOI:** 10.1186/s12886-018-0779-1

**Published:** 2018-05-21

**Authors:** Ye He, Xin-jun Ren, Bo-jie Hu, Wai-Ching Lam, Xiao-rong Li

**Affiliations:** 1 Department of Retina, Tianjin Medical University Eye Hospital, 251 Fukang Road, Tianjin, 300384 China; 2 Department of Ophthalmology, The University of Hong Kong, Hong Kong, China

**Keywords:** Diabetic macular edema, Ozurdex, Dexamethasone implant, Anti-VEGF, Meta-analysis

## Abstract

**Background:**

This meta-analysis evaluated the effectiveness and safety of dexamethasone (DEX) implant and intravitreal anti-vascular endothelial growth factor (VEGF) treatment for diabetic macular edema (DME).

**Methods:**

The PubMed, Embase, clinicaltrials.gov website and Cochrane Library databases were comprehensively searched for studies comparing DEX implant with anti-VEGF in patients with DME. Best-corrected visual acuity (BCVA), central subfield thickness (CST) and adverse events were extracted from the final eligible studies. Review Manager (RevMan) 5.3 for Mac was used to analyze the data and GRADE profiler were used to access the quality of outcomes.

**Results:**

Based on four randomized clinical trials assessing a total of 521 eyes, the DEX implant can achieve visual acuity improvement for DME at rates similar to those achieved via anti-VEGF treatment (mean difference [MD] = − 0.43, *P* = 0.35), with superior anatomic outcomes at 6 months (MD = − 86.71 μm, *P* = 0.02), while requiring fewer injections, in comparison to anti-VEGF treatment. Although the mean reduction in CST did not showed significant difference at 12 months (MD = − 33.77 μm, *P* = 0.21), the significant in BCVA from baseline to 12 months supported the anti-VEGF treatment (MD = − 3.26, *P* < 0.00001). No statistically significant differences in terms of the serious adverse events. However, use of the DEX implant has higher risk of intraocular pressure elevation and cataract than anti-VEGF treatment.

**Conclusions:**

Compared with anti-VEGF, DEX implant improved anatomical outcomes significantly. However, this did not translate to improved visual acuity, which may be due to the progression of cataract. Therefore, the DEX implant may be recommended as a first chioce for select cases, such as for pseudophakic eyes, anti-VEGF-resistant eyes, or patients reluctant to receive intravitreal injections frequently.

**Electronic supplementary material:**

The online version of this article (10.1186/s12886-018-0779-1) contains supplementary material, which is available to authorized users.

## Background

Macular edema (ME) is not an independent disease, but a common phenomenon in various retinal diseases in which fluid and protein accumulate in the extracellular space within the retina [1, 2]. Diabetic macular edema (DME) is macular thickening secondary to diabetic retinopathy (DR) that may be present in any of the stages of this disease, although it manifests more commonly in the non-proliferative diabetic retinopathy stage. In patients with DR, aged 20 to 79 years, the global prevalence for DME is 6.8% [3]. The prevalence of DME is reported to be related to the duration of the diabetes [4, 5]. DME is the foremost cause of central vision loss, and even blindness, and has a great influence on life quality of patients. Thus, reduction of ME may be associated with improved vision.

However, the treatment of DME remains controversial among vitreoretinal specialists. In addition to glycemic control, a variety of treatment alternatives exist for patients presenting with DME, including focal or grid photocoagulation, which has been the standard therapy since the 1970s, and more recently, intravitreal injection (anti-VEGF or corticosteroids) has been applied to DME, and vitrectomy in patients with DME with vitreomacular traction. As understanding of the pathophysiology of DME has improved, focal or grid photocoagulation is no longer the first choice for the treatment of DME. Treatment now targets the causal factor specifically, VEGF. To date, anti-VEGF drugs, including aflibercept, ranibizumab, and bevacizumab, have been proven in many clinical trials with efficacy for DME. The RESOLVE [6], RISE/RIDE [7, 8], and READ-2 [9] studies have all shown that ranibizumab is a good choice for the treatment of DME. The BOLT [10] randomized trial showed that bevacizumab is superior to laser monotherapy for persistent center-involving clinically significant macular edema.

Anti-VEGF drugs is still the first-line for treating DME, but also may impose a significant burden for patients who either do not have good respond to anti-VEGF or have recurrent ME, require frequent anti-VEGF injections [11]. Indeed, a study by the Diabetic Retinopathy Clinical Research Network (DRCR. Net), Protocol I revealed that more than 40% of ranibizumab-treated eyes still had CST ≥ 250 μm at 2 years post-treatment. The limited visual gains or resolution of DME in those naive patients are believed to be related to the pathophysiology of DME. Although the contribution of VEGF to the development of DME is indisputable, the role of other non-VEGF pathways has also been considered. Many studies have demonstrated that the inflammation is involved in the DR progression. With the increased recognition of the role of inflammation in the development of DME, sustained-release implants of steroids have shown good anatomical benefit and have also, to some extent, reduced the number of intravitreal injections that is needed in most cases.

Dexamethasone, it has the highest relative clinical efficacy of any corticosteroid applied to ophthalmological practice, and exerts its multiple-effects via its influence on multiple signal transduction pathways. The 0.7 mg intravitreal DEX implant is a biodegradable solid polymer drug-delivery system (Ozurdex®, Allergan, Inc.), which uses the following characteristic mode of drug release by diffusion, in a biphasic fashion: an initial high-concentration phase and a second low-concentration phase, which facilitates continued efficacy of the treatment for up to 6 months [12]. The U.S. Food & Drug Administration (FDA) first approved the Ozurdex for retinal vein occlusion-induced ME treatment in 2009. It was then approved for non-infectious uveitis treatment. In 2014, the FDA and most European countries approved Ozurdex for the treatment of DME, based on results of the MEAD study [13]. Studies of treatment of DME have demonstrated that Ozurdex may be an alternative treatment for patients who do not have good respond to serial anti-VEGF injections or in recalcitrant cases [14–19]. In addition, Ozurdex may be considered as primary treatment for DME [20].

To date, no systematic review has discussed the therapeutic effect and safety of intravitreal anti-VEGF versus DEX implant in DME. We performed a systematic review and meta-analysis to quantify the effect of these two treatments on BCVA and CST in DME. Additionally, we report the adverse events described with these therapies.

## Methods

### Search strategy

**Fig. 1:**
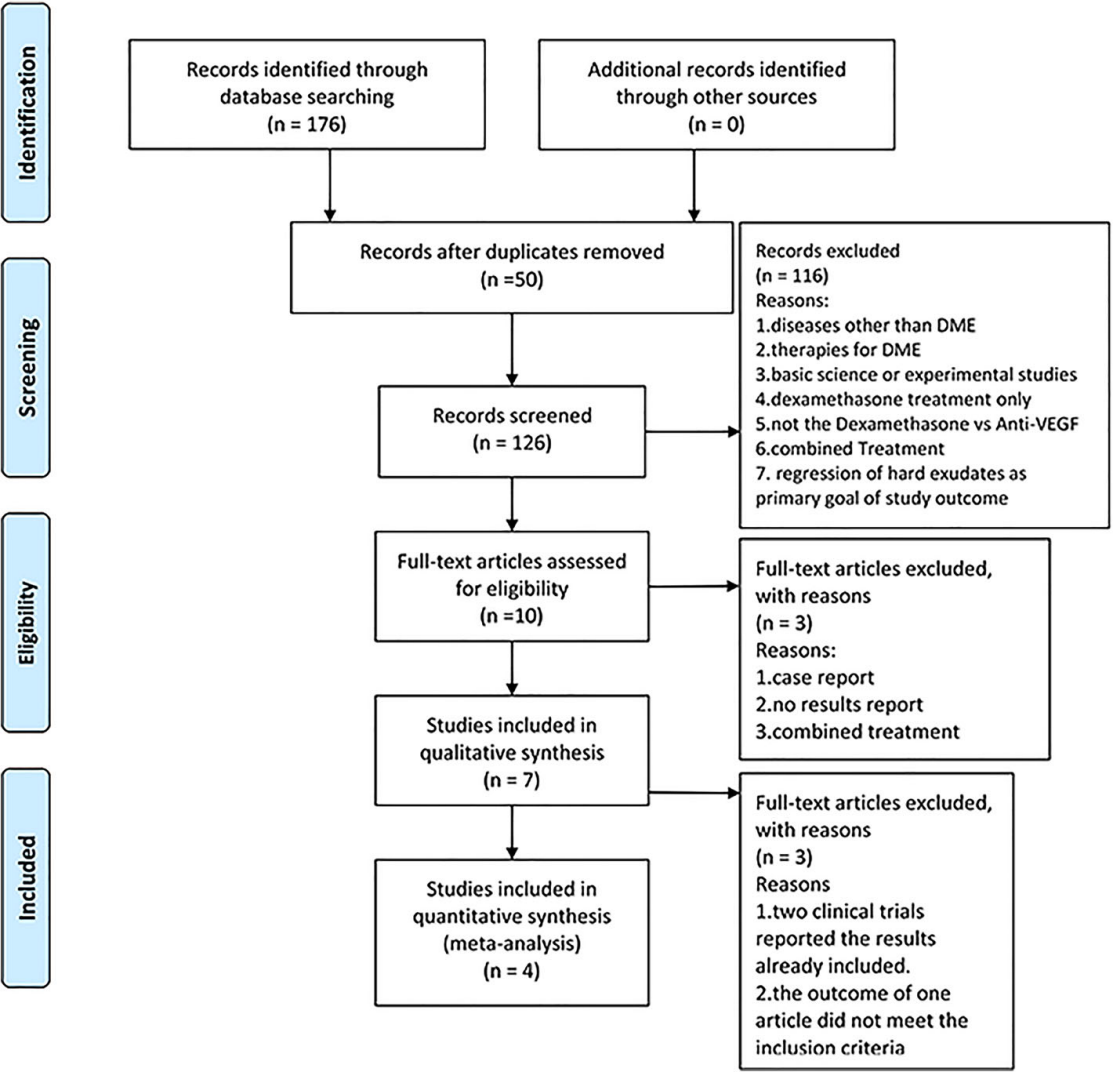
Flow chart of the literature search

The study was conducted in accordance with Cochrane Handbook for Systematic Reviews and Meta-Analysis (PRISMA) guidelines (Additional file 1) [21]. The following databases were screened: including PubMed, Embase, clinicaltrials.gov, and the Cochrane Library, up to August 2017 (Additional file 2, Table 1). Keywords, including macular edema, dexamethasone intravitreal implant, dexamethasone, anti-VEGF, and Ozurdex were used to maxmise the search accuracy. The literature selections are shown in the PRISMA flow diagram in Fig. 1.

**Table 1:** Summary of the characteristics of the included studies

Study	Place	Conditions	Participants numbers	Interventions details	Total number of treatments	Age (years)	Female sex, no. (%)	BCVA at baseline	CST/CMT (μm) at baseline	Follow-up duration (months)
Gillies et al. 2014 < The BEVORDEX Study> [23]	Australia	DR	DEX: 46 IVB: 42	DEX: 0.7 mg every 16 weeks + PRN IVB: 0.5 mg every 4 weeks + PRN	DEX: 2.7 IVB: 8.6	DEX: 61.4 ± 9.0 IVB: 62.2 ± 10.5 (*P* = 0.71)	DEX: 16 (35%) IVB: 16 (38%) (*P* = 0.83)	DEX: 55.5 ± 12.5 IVB: 56.3 ± 11.9 (*P* = 0.75)	DEX: 474.3 ± 95.9 IVB: 503 ± 140.9 (*P* = 0.38)	12
Allergan 2015 [27]	Multiple countries: Belgium, Denmark, France, Germany, Israel, Italy, Netherlands, Portugal, South Africa, Spain, United Kingdom, United States	DR	DEX: 181 IVR: 182	DEX: 0.7 mg on Day 1, Month 5, and Month 10 IVR: 0.5 mg into the study eye on Day 1. Patients may receive additional injections on a monthly basis, as needed, for disease progression	NA	NA	DEX: 69 (38%) IVR: 66 (36%)	DEX: 60.2 ± 9.74 IVR: 60.4 ± 9.34	DEX: 465.1 ± 136.09 IVR: 471.2 ± 139.51	12
Shah et al. 2016 [24]	Indiana, United States	DR	DEX: 27 IVB: 23	DEX: 0.7 mg given every 3 months over 6 month period with a maximum of 3 injections IVB: 1.25 mg given monthly during a 6 month period	DEX: 2.7 ± 0.5 IVB: 7.0 ± 0.2 (*P* < 0.001)	DEX: 65 ± 11 IVB: 61 ± 9 (*P* = 0.209)	DEX: 15 (56%) IVB: 10 (44%) (*P* = 0.571)	DEX: 59 ± 12 IVB: 59 ± 13 (*P* = 0.770)	DEX: 458 ± 100 IVB: 485 ± 122 (*P* = 0.508)	7
Gallemore et al. 2017 [26]	California, United States	DR	DEX: 10 IVB: 10	DEX: Ozurdex, 0.7 mg given at initial visit and at month 4 (visit 5) IVB: 1.25 mg given at initial visit and Q1 month for a total of 5 treatments	NA	DEX: 63.9 ± 1.8 IVB: 61.2 ± 2.9	DEX: 5 (50%) IVB: 3 (30%)	DEX: 67.8 ± 3.8 IVB: 71.9 ± 2.9	DEX: 385.9 ± 43.0 IVB: 341.5 ± 11.3	6

*NA* not available, *PRN* pro re nata

### Inclusion and exclusion criteria

Studies were regarded eligible if they accord with the following criterias: (1) the study population included patients with DME; (2) the DEX implant (Ozurdex) was included as an intervention; (3) there was a comparison between the DEX implant (Ozurdex®) and anti-VEGF. Through our analysis of the studies, we determined the following primary outcomes. First, the mean BCVA and mean improvement from baseline in BCVA [time points: baseline, 6 months, and 12 months]. BCVA was obtained using the Early Treatment Diabetic Retinopathy Study (ETDRS). Second, the mean CST and mean change from baseline in CST or foveal thickness, and central macular thickness (CMT) was demonstrated on optical coherence tomography (OCT) [time points: baseline, 6 months, and 12 months]. Additional outcomes collected included the following: 1) total number of serious adverse events (SAEs) at the end of each study; 2) elevation of intraocular pressure (IOP>21 mmHg, required glaucoma agents for IOP control, or IOP elevation by at least 5 mmHg from baseline at any follow-up visit; 3) the number of cataracts; 4) the mean number of intravitreal injections; and 5) the study design should be randomized controlled trials (RCTs).

We designed the study to have no limitations on dose. Patients taking bevacizumab and ranibizumab were placed in the anti-VEGF group. The two authors, Ye He and Bo-jie Hu assessed all eligible studies independently. A consensus was reached if there were any cases of disagreement. The exclusion criteria included studies with insufficient data, non-RCTs, case reports, review articles.

### Data extraction and risk of bias assessment

The relevant data from the articles were extracted by two reviewers (Ye He and Bo-jie Hu) independently, using a standard data extraction form. The extracted data included the first author(s) or the information provider, publishing date, study design, sample size, geographical location of the research, interventions details, age, sex, outcomes and follow-up periods. We emailed the corresponding authors of the studies for which we had unanswered questions to ensure completeness of our study, and to acquire incomplete and missing data. Data are showed in the format: mean ± standard deviation (SD). We used the formula that SD = SE*√N) to calculate SD if the data was reported as standard error (SE). Afterwards, we used Get Data software to estimate the mean and the SD from the reported graph. The Cochrane Collaboration’s tool was applied to assess the risk of bias in each study based on the Cochrane Handbook.

### Statistical analysis

RevMan 5.3 was applied to integration collected data statistics and analysis. The mean difference (MD) and risk ratio (RR) were used to assess continuous variable outcomes and dichotomous outcomes with a 95% confidence interval [CI], respectively. The heterogeneity of studies was accessed using the chi-square test based on the values of P and I^2^. The random-effects model was applied for the meta-analysis. I^2^ results between 50 and 100% represented substantial heterogeneity. *P* values < 0.05 were considered statistically significant.

### Quality of the evidence

The evidence quality of all included outcomes was evaluated based on the GRADE system [22]. Initially, RCTs were regarded as high-quality evidence for the estimation of study effects. Factors such as risk of bias, imprecision and inconsistency of results etc., all of which can result in rating down the quality of evidence for specific outcomes, reduced the level of confidence in estimating the study effects. The GRADE evidence was divided into the four categories (High, Moderate, Low and Very low-quality evidence).

## Results

### Search results

A total of 176 potential records up to August 2017 were identified with the electronic-based search (PubMed = 83, Embase = 52, clinicaltrials.gov=38, and the Cochrane Library = 3). After eliminating 50 duplicates, a total of 126 potentially eligible studies were retrieved. After reading the title and abstract, 116 of these studies were excluded. We further excluded three studies after reading the full text due to ineligible for criteria. Among the 7 studies included in the qualitative synthesis, two clinical trials (ClinicalTrials.gov identifier: NCT01298076; NCT02036424) were duplicates, because the data had already been published. Among these published articles [23–25], the outcome of one article did not meet the inclusion criteria [25]. After looking through all eligible studies, four studies comprising 521 study eyes were used in our meta-analysis (Fig. 1) [23, 24, 26, 27].

**Fig. 2:**
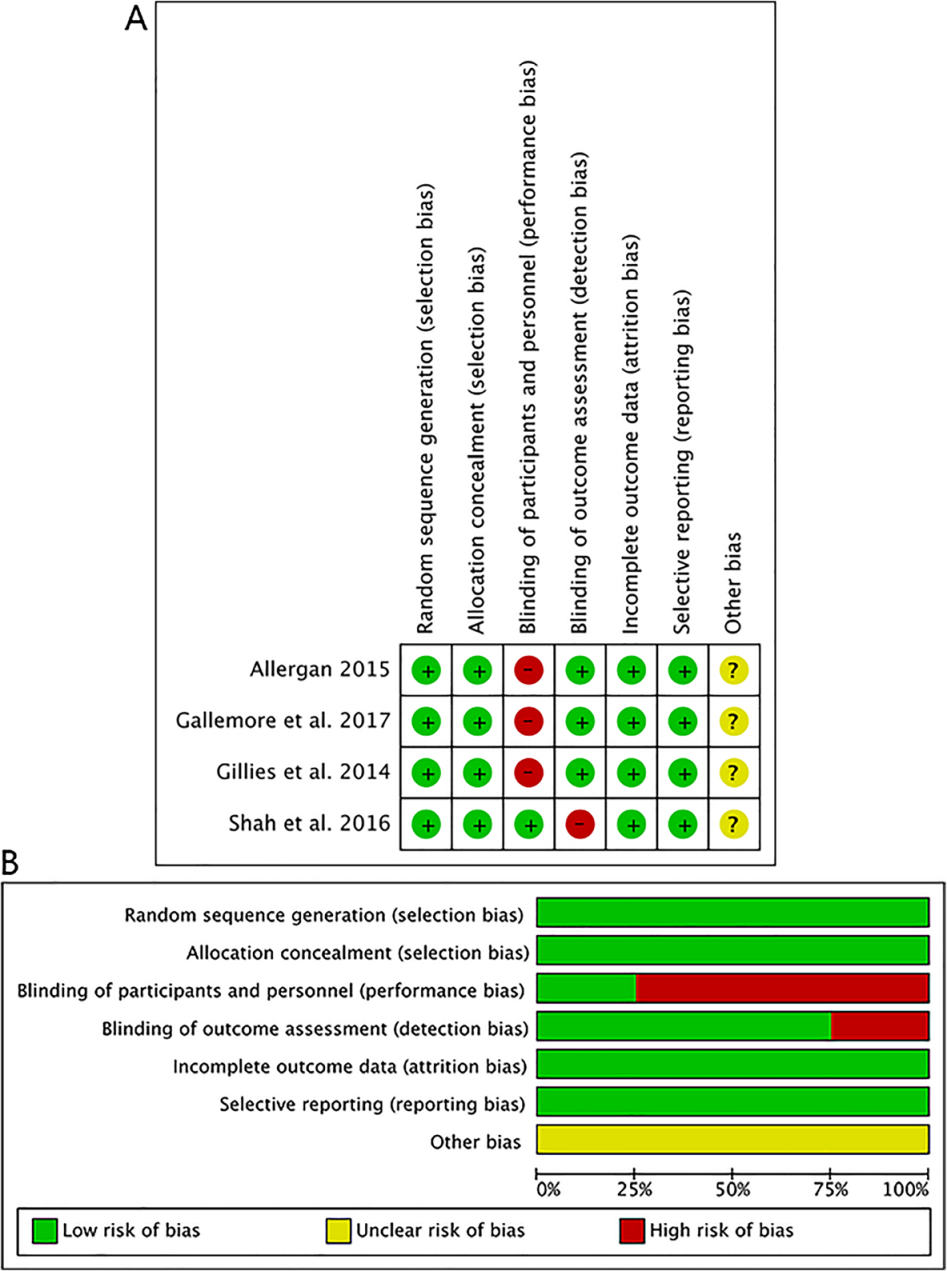
Assessment of the risk of bias in included studies. **a** Risk of bias summary: review authors’ judgements about each risk of bias item for each included study. +: low risk of bias; −: high risk of bias;?: unclear risk of bias. **b** Risk of bias graph: review authors’ judgements about each risk of bias item presented as percentages across all included studies

All of included studies were RCTs and the characteristics of these studies are summarized in Table 1. The sample size of the four studies ranged from 20 to 363. Two studies were published in 2014 and 2016. Two studies reported their results online and verified the results in January 2015 and April 2017, respectively. In all included studies, the dose of the DEX implant was the same. However, in the study by Shah et al., dexamethasone (0.7 mg) was given every 3 months instead of every 4 months as in the other included studies. Among them, Gillies et al., Shah et al., and Gallemore et al. performed intravitreal bevacizumab (IVB) injection, while Allergan study used intravitreal ranibizumab (IVR) injection. The risk of bias assessment and the results of the GRADE evidence are presented in Fig. 2 and Additional file 2: Table S2, respectively.

### Meta-analysis results

#### Mean BCVA at 6 months

**Fig. 3:**
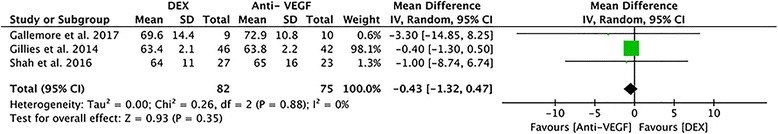
A forest plot diagram showing the mean BCVA and the associated 95% CI, comparing DEX with Anti-VEGF treatment at 6 months

Data from three studies assessing 157 eyes (82 eyes with DEX treatment, 75 eyes with anti-VEGF treatment) reported the BCVA at 6 months. No difference in the treatment effect on BCVA at 6 months between the two treatment arms; the MD in visual acuity of the three trials was − 0.43 (95% CI: -1.32 to 0.47, *P* = 0.35, Fig. 3). No statistical heterogeneity was found (chi^2^ = 0.26, *P* = 0.88, I^2^ = 0%).

#### Mean change in BCVA at 6 months and 12 months

**Fig. 4:**
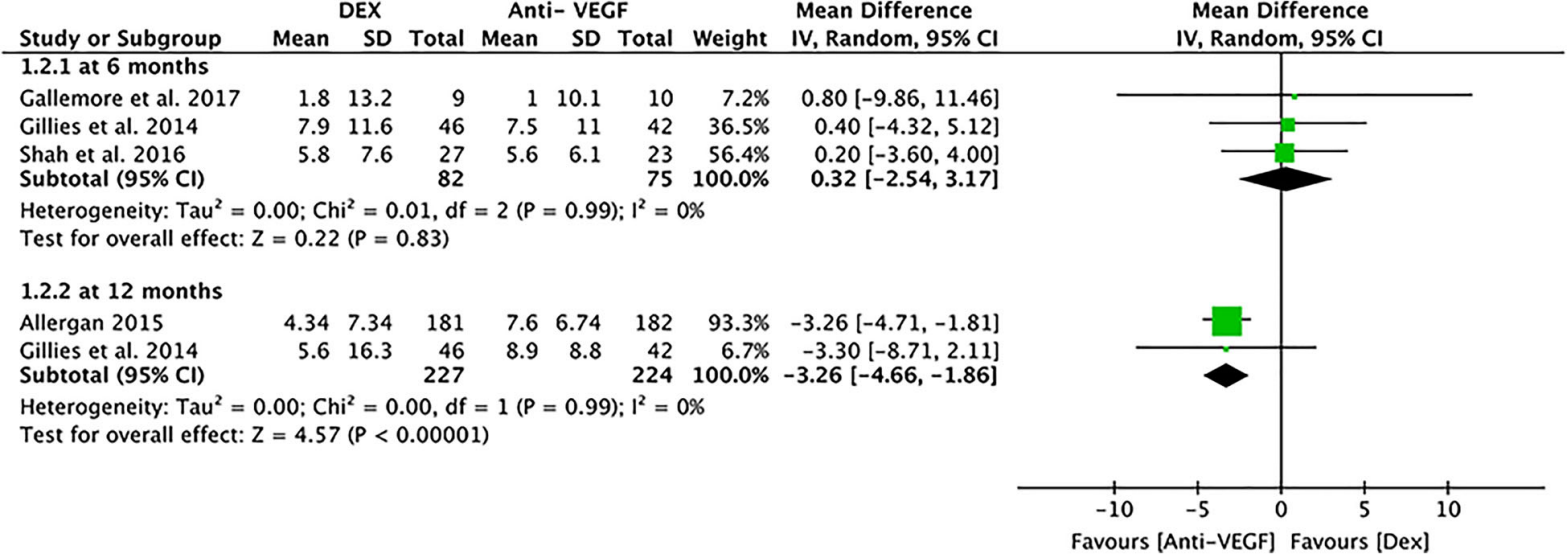
A forest plot diagram showing the mean change in BCVA and the associated 95% CI, comparing DEX with Anti-VEGF treatment at 6 months and 12 months

At 6 months, data from three studies assessing 157 eyes reported the an improvement in BCVA from baseline. Because the clinical effect in 6 months was similar to that in 7 months, we used the data from month 7 to represent month 6 in the study by Shah et al. [24]. The DEX group reported a similar mean change in BCVA from baseline compared with the anti-VEGF group (MD = 0.32; 95% CI, − 2.54 to 3.17; *P* = 0.83; Fig. 4), and no heterogeneity was found (*P* = 0.99, I^2^ = 0%). A meta-analysis at 12 months was conducted using two studies of 451 eyes to assess the improvement in BCVA from baseline. Statistically significant differences were discovered between the DEX implant and anti-VEGF treatment groups, in favor of the anti-VEGF group (MD = − 3.26, 95% CI: -4.66 to − 1.86, *P* < 0.00001; Fig. 4) and no heterogeneity was found (*P* = 0.99, I^2^ = 0%).

#### Mean CST at 6 months

**Fig. 5:**
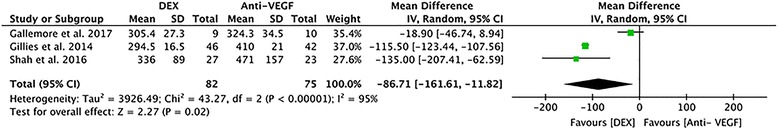
A forest plot diagram showing the mean CST and the associated 95% CI, comparing DEX with Anti-VEGF treatment at 6 months

Three studies of 157 eyes included data on CST at 6 months after the initial treatment. Meta-analysis demonstrated that all studies except that by Gallemore et al. [26] showed a marked reduction in CST from baseline in the DEX group. The MD for all studies at 6 months was statistically significant (MD = − 86.71 μm, 95% CI: − 161.61 to − 11.82, *P* = 0.02) in favor of DEX treatment over anti-VEGF treatment, and showed high heterogeneity (*P* < 0.00001, I^2^ = 95%; Fig. 5).

#### Mean change in CST at 6 months and 12 months

**Fig. 6:**
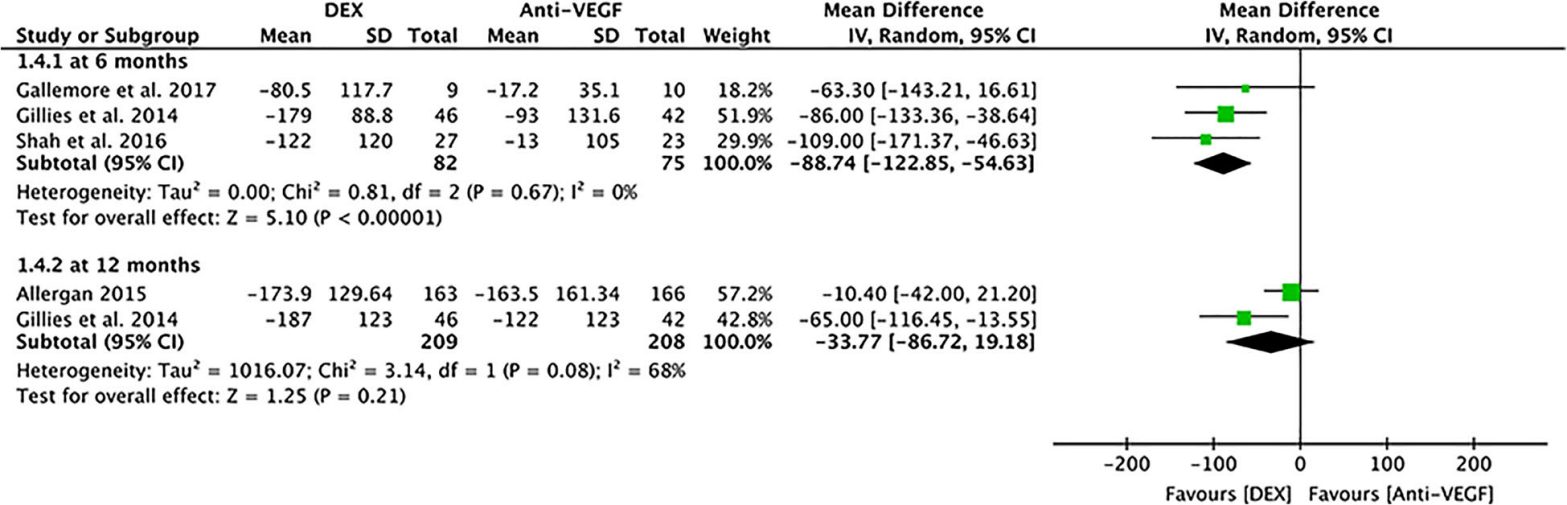
A forest plot diagram showing the mean change in CST and the associated 95% CI, comparing DEX with Anti-VEGF treatment at 6 months and 12 months

Data from three studies assessing 157 eyes reported the mean change from baseline to 6 months in terms of the reduction of CST was significantly greater in the DEX groups (MD = − 88.74, 95% CI: -122.85 to − 54.63, *P* < 0.00001, Fig. 6). However, this superiority was no longer observed at 12 months. Data from two studies assessing 417 eyes at 12 months, with a combined mean difference in CST of − 33.77 μm, did not show statistically significant differences (95% CI: -86.72 to − 19.18, *P* = 0.21), and showed a large amount of heterogeneity between the two studies (*P* = 0.08, I^2^ = 68%, Fig. 6).

#### Total serious adverse events

**Fig. 7:**
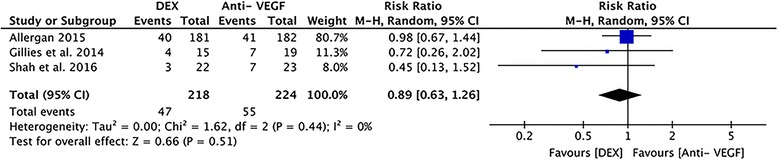
A forest plot diagram showing the total serious adverse events

All four studies reported complications during the follow-up period, such as increased IOP, cataract, and vitreous hemorrhage. In these studies, the study by Gallemore et al. reported no serious adverse events during the follow-up period. The total SAEs reported by the three studies are presented in the forest plot (Fig. 7). Analysis of the available data demonstrated a lower incidence of serious adverse events in the DEX arm (RR = 0.89), with no heterogeneity (*P* = 0.44, I^2^ = 0%), however the differences were not statistically significant (95% CI: 0.63 to 1.26; *P* = 0.51).

#### Elevation of IOP

**Fig. 8:**
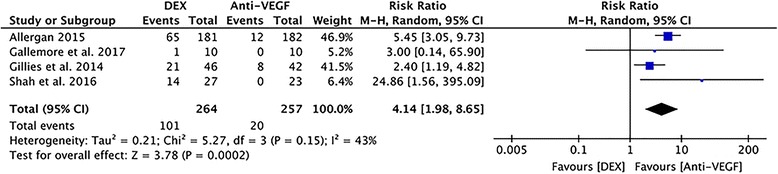
A forest plot diagram showing the elevation of IOP

All cases demonstrated increased IOP after injection of DEX/anti-VEGF; this was mostly controllable by medication or surgery. Low heterogeneity was detected between studies (I^2^ = 43%, *P* = 0.15, Fig. 8). Analysis using a random-effects model demonstrated a statistically significant difference between DEX and anti-VEGF treatment (RR = 4.14; 95% CI: 1.89 to 8.65; *P* = 0.0002).

#### Adverse events: cataract

**Fig. 9:**
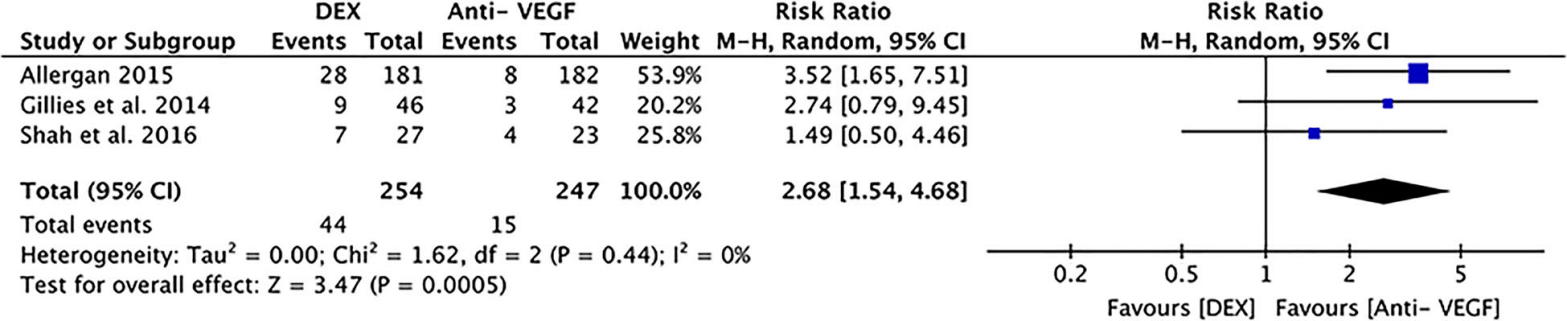
A forest plot diagram showing the adverse events: cataract

Three studies involving 501 eyes reported post-operative cataract. Statistically significant difference was founded between the DEX and anti-VEGF groups (RR =2.68, 95% CI: 1.54 to 4.68, *P* = 0.0005), without heterogeneity (*P* = 0.44, I^2^ = 0%, Fig. 9).

### Mean number of intravitreal injections

In the study by Shah et al. [24], more injections were required in the IVB group (7.0 ± 0.2) compared to the DEX group (*P* = 0.001) over a follow-up period. Similarly, no statistically significant difference in BCVA according to Gillies et al., but a lower treatment frequency was required for the DEX implant (mean number: 2.7), which was an obvious advantage over IVB treatment (mean number: 8.6) [23].

## Discussion

In our study, we evaluated four RCTs to evaluate the efficacies of DEX implants and anti-VEGF in the treatment of DME. We found that both DEX implant and anti-VEGF could achieve significant functional and anatomical improvement during early treatment. We did not find statistically significant differences in terms of BCVA and with no statistical heterogeneity between these two treatment arms at 6 months. However, the anti-VEGF group revealed significant improvement in BCVA at 12 months, compared to the DEX implant group, and without statistical heterogeneity. These findings are in line with the Protocol I in DRCR.net, whereby the ranibizumab group had better visual acuity than the triamcinolone group.

Even with good treatment effects, repeated injections carry increased risks, such as infectious endophthalmitis, intraocular inflammation, and even stroke or myocardial infarction [28]. Therefore, anti-VEGF treatment may not be a good therapy for all patients. DME has been shown to be a complex multifactorial disease; in addition to VEGF, inflammation may be another pathophysiological feature of these treatment-naive patients. Diabetics are found to have high concentrations of pro-inflammatory cytokines, such as interleukin 6 (IL-6), IL-1β, tumor necrosis factor α (TNFα), and intracellular adhesion molecule (ICAM)-1. All of these cytokines induce retina with persistent chronic inflammation, which leads to leukostasis, increased vascular permeability and dysfunction of the blood-retinal barrier (BRB) [29, 30]. Additionally, according to an investigation by Jonas et al. [31], DME was shown to be related to elevated cytokines in the aqueous humor or vitreous, such as ICAM-1, IL-6, transforming growth factor beta (TGF-β) and monocyte chemotactic protein 1 (MCP-1). Among the cytokines, ICAM-1 is closely related with diabetes characteristics. Thus, treatment principally aims to block the effect of these two pathogenic pathways. Corticosteroids have anti-inflammatory, anti-permeability, and angiostatic effects when treating DME [32]. Thus, DEX implant could be a better alternative for DME.

Our analysis demonstrated that DEX implant treatment could significantly reduce CST at 6 months, compared to anti-VEGF treatment. Unfortunately, this effect can not last until 12 months. This can be explained by the characteristics of the DEX implant. There are two phases in the DEX implant treatment. Higher concentrations of DEX are found in the initial phase, followed by lower concentrations in the final phase. The drug reaches its peak efficacy less than two months after administration. Afterwards, the drug continues to provide treatment, but, at lower levels in months 2–6 [12]. Regarding heterogeneity, we could not perform a subgroup analysis to interpret the potential source of heterogeneity, due to the limited studies. We suspect that, at 6 months, the heterogeneity mainly derives from the small sample size of the study by Gallemore et al. Moreover, the results of CST may be affected by other factors, including initial retinal thickness, the dosage of bevacizumab, and previous treatment (laser or anti-VEGF). The mean change in CST at 12 months showed heterogeneity mainly caused by the type of anti-VEGF drugs used. In the Allergan 2015 study [27], patients received 0.5 mg ranibizumab. However, in the BEVORDEX study [23], 0.5 mg bevacizumab was given to the patients. It has been reported that ranibizumab and bevacizumab do not differ in terms of functional outcomes, but that ranibizumab was more effective in terms of CST reduction [33]. Factors such as race, dosage, age, and baseline states may have attributed to heterogeneity. To some extent, these factors were unavoidable. In terms of CST, at 6 months, Ozurdex showed superior reduction in CST, which indicates that patients with DME can achieve superior anatomical outcomes with fewer injections. Similar to our results, the results from the MEAD study evaluating the DEX implant showed that a mean number of 4.1 DEX treatments were administered after 3 years of follow-up. Compared to the sham group, the DEX group acquired a CST reduction of 112 μm, nearly three times than the sham group (42 μm).

Notably, the improved anatomical outcomes in our data did not translate to improved visual acuity outcomes. Results from other clinical trials were consistent with the four studies included here. In the CHROME study [34], significant reduction in retinal thickness were occurred in the Ozurdex group: 190.9 ± 23.5 μm for DME (*P* < 0.0001). The greatest mean improvements in BCVA in terms of number of lines of vision observed in the eyes with DME (0.7 ± 0.5; *P* > 0.05). Similarly, compared to monthly IVB monotherapy (− 30 μm), the combination therapy with DEX and bevacizumab led to a significantly greater change of CST (− 45 μm) in the study by Maturi et al.; nervertheless, visual acuity improvement was not statistically significant [35]. The limited visual gains in these patients may have been related to the duration of macular edema, neural damage, retinal pigment epithelium changes, and subretinal fibrosis caused by chronic macular edema prior to treatment, as well as the result of structural damage from repeated macular laser therapy, and the natural course of DR [7].

Additionally, we suspect that it may be due to DEX-induced progression of cataract, based on previous studies. In the DRCR Protocol I, when controlling for cataract formation, in pseudophakic eyes, the group that received triamcinolone observed similar BCVA results to the ranibizumab group. Similar to this study, the CHROME study also showed that the pseudophakic eyes with DME acquired a average gain of 1.4 ± 0.5 lines, but a average loss of 0.6 ± 0.6 lines was shown in phakic eyes [34]. These results are also in agreement with those of the BEVORDEX study, which found exhibited no significant effect for pseudophakic eyes. Compared to 10.4 letters in the dexamethasone group, the mean change in BCVA for the bevacizumab group was 7.7 letters (*P* = 0.47). Moreover, visual acuity may be affected by post-operative complications, such as Irvine−Gass syndrome or cataract secondary to pars plana vitrectomy prior to DEX or anti-VEGF treatment.

In addition to having different efficacies for treating DME, DEX implants and anti-VEGF injection are associated with varying degrees of increased risk of systemic and/or local complications over the period of treatment. The systemic adverse event rate has been reported to be higher with anti-VEGF treatment in some clinical trials [36]. In the study by Avery et al., the increased potentially cerebrovascular accidents may be link to anti-VEGF treatment, especially after two years therapy [37]. Similar to previous studies, our data demonstrated that a lower incidence of SAEs in the DEX implant group, but this was not statistically significantly different between the DEX and anti-VEGF group. These results suggest that we should be cautious in using anti-VEGF in patients with myocardial infarction and stroke [38]. The deterioration of hypertension was the most frequent systemic adverse event encountered in the BEVORDEX study. Other adverse events, such as cardiac disorders, also occurred in the studies included in this meta-analysis, except in the study by Gallemore et al. High IOP and secondary cataract are the most common ocular adverse events of DEX implants. Our meta-analysis agrees with these findings, which demonstrated a statistically significant difference between the two groups in terms of increased IOP and cataract. The groups receiving DEX had a higher risk of a rise in IOP and cataract progression than the anti-VEGF groups for DME. This suggests that the ophthalmologist should take care when using DEX implants in patients with high IOP or in young patients with a clear lens.

The relative benefits and costs should also be considered when applying therapies. In terms of cost-effectiveness, intravitreal corticosteroid injections are relatively cheaper than anti-VEGF therapies, although this is not true for ranibizumab. One study calculated the amount of money (USD) saved per line of visual acuity improvement for each of the various therapies as follows: DEX implant—$5666, bevacizumab—$1329–$2246, and ranibizumab—$11,372–$11,609. In addition, the dollars per quality-adjusted life year for these therapies were as follows: DEX implant—$9446, bevacizumab—$2013–$4260, and ranibizumab—$19,251–$23,119 [39].

Currently, dexamethasone delivery systems and anti-VEGF therapies have a positive effect on the course of DME. However, these two different types of drugs have different pharmacological properties and side-effect characteristics. Given the results of clinical trials and the pathophysiology of DME, Ozurdex is considered to be the preferred treatment for patients who have chronic DME and are anti-VEGF-resistant, as an alternative to switching between anti-VEGF drugs [40, 41]. Ozurdex may be recommended as a first choice for the following cases: 1. pseudophakic eyes, or patients who are under consideration for cataract surgery in the near future; 2. patients who are anti-VEGF-resistant [42]; 3. patients who have a history of cardiovascular and cerebrovascular diseases [42]; 4. post-vitrectomy patients [18]; 5. patients without a high IOP risk at baseline; 6. patients who are reluctant to receive frequent injections. In all cases, the IOP should to be monitored frequently. Reinjection of Ozurdex can be considered after approximately 3−6 months if remains evidence of impaired vision and residual ME.

Our study was limited by the following factors: (1) We only included four studies assessing a total of 521 eyes. (2) The clinical trails duration was quite short in some of the that were included, and thus we may have underestimated the drug-induced adverse events. (3) Heterogeneity was inevitable due to the different regimens of anti-VEGF therapies used. Previous studies indicated that the efficacy of aflibercept, ranibizumab or bevacizumab for DME was different but the relative efficacy depended on baseline BCVA. At the 1-year follow-up, aflibercept exhibited some advantage over bevacizumab and ranibizumab, especially among patients with an initial baseline BCVA letter score of less than 69. However, ranibizumab and bevacizumab did not show significant differences [28, 33]. A previous meta-analysis study also confirmed that bevacizumab and ranibizumab did not differ in terms of BCVA, but ranibizumab was more effective in terms of CST reduction with a low-certainty of evidence [43]. To reinforce the validity of our meta-analysis, clinical trials comparing the 3 anti-VEGF agents with the DEX implant as well as extended follow-up trials should be conducted in the future.

## Conclusions

In summary, this meta-analysis of data from four randomized clinical trials revealed that despite some ocular adverse events, DEX-treated eyes had relatively superior anatomic outcomes compared with anti-VEGF, and showed similar rates of vision improvement, while requiring fewer injections, especially in pseudophakic patients. However, considering for the restrictions of indications, the DEX implant may not be recommended as a first-line therapy for DME. In the future, randomization of these treatments would allow a definite conclusion about whether switching to a DEX implant is more beneficial rather than anti-VEGF treatment. Additionally, new treatments (monotherapy or combined therapy) should be investigated to optimize clinical efficacy and reduce side-effects.

## Additional files

Additional file 1: PRISMA 2009 checklist. (PDF 316 kb)

Additional file 2: **Table S1.** Search strategy on PubMed; **Table S2.** GRADE of the evidence. (PDF 355 kb)

## Abbreviations

BCVA: Best corrected visual acuity
CI: Confidence interval
CMT: Central macular thickness
CST: Central subfield thickness
DEX implant: Dexamethasone intravitreal implant
DRCR. Net: Diabetic Retinopathy Clinical Research Network
ETDRS: Early Treatment Diabetic Retinopathy Study
FDA: Food & Drug Administration
IVB: Intravitreal bevacizumab
IVR: Intravitreal ranibizumab
MD: Mean difference
ME: Macular edema
NA: Not available
OCT: Optical coherence tomography
PRN: Pro re nata
RCTs: Randomized controlled trials
RevMan: Review Manager
RR: Risk ratio
SAEs: Serious adverse events
SD: Standard deviation
SE: Standard error
VEGF: Vascular endothelial growth factor
