# Supplementary material for: A meta-analysis of the effect of a dexamethasone intravitreal implant versus intravitreal anti-vascular endothelial growth factor treatment for diabetic macular edema
Source: BMC Ophthalmol. 2018 May 21;18:121. doi: 10.1186/s12886-018-0779-1 (PMC5963018; doi:10.1186/s12886-018-0779-1)
Supplement: Supplementary file 2 — Table S1. Search strategy on PubMed; Table S2. GRADE of the evidence. (PDF 355 kb) [file 12886_2018_779_MOESM2_ESM.pdf]

Table S1

Search strategy on PubMed

#1 Search (((macular edema) AND intravitreal dexamethasone implant) AND anti-VEGF) AND diabetes

#2 Search (((macular edema) AND diabetes) AND dexamethasone) AND anti-VEGF

#3 Search (((macular edema) AND diabetes) AND anti-VEGF) AND Ozurdex

Table S2 GRADE of the evidence

Quality assessment							No of patients		Quality	Importance
No of studies	Design	Risk of bias	Inconsistency	Indirectness	Imprecision	Other considerations	OUTCOME	Control		
**Mean BCVA at 6 months (follow-up mean 6 months; measured with: ETDRS; Better indicated by lower values)**										
3	randomised trials	serious[1]	no serious inconsistency	no serious indirectness	serious[2]	reporting bias[3]	82	75	⊕OOO VERY LOW	CRITICAL
**Mean change in BCVA at 6 months and 12 months - at 6 months (follow-up mean 6 months; measured with: ETDRS; Better indicated by lower values)**										
3	randomised trials	serious[1]	no serious inconsistency	no serious indirectness	serious[2]	reporting bias[3]	82	75	⊕OOO VERY LOW	CRITICAL

**Mean change in BCVA at 6 months and 12 months - at 12 months (follow-up 12 months; measured with: ETDRS; Better indicated by lower values)**										
2	randomised trials	no serious risk of bias	no serious inconsistency	no serious indirectness	no serious imprecision[2]	reporting bias[3]	227	224	⊕⊕⊕O MODERATE	CRITICAL
**Mean CST at 6 months (follow-up 6 months; measured with: OCT; Better indicated by lower values)**										
3	randomised trials	serious[1]	serious[4]	no serious indirectness	serious[2]	reporting bias[3]	82	75	⊕OOO VERY LOW	CRITICAL
**Mean change in CST at 6 months and 12 months - at 6 months (follow-up 6 months; measured with: OCT; Better indicated by lower values)**										
3	randomised trials	serious[1]	no serious inconsistency	no serious indirectness	serious[2]	reporting bias[3]	82	75	⊕OOO VERY LOW	CRITICAL
**Mean change in CST at 6 months and 12 months - at 12 months (follow-up 12 months; measured with: OCT; Better indicated by lower values)**										
2	randomised trials	no serious risk of bias	serious[4]	no serious indirectness	no serious imprecision	reporting bias[3]	209	208	⊕⊕OO LOW	CRITICAL
**Total serious adverse events (follow-up 12 months)**										
3	randomised trials	serious[1]	no serious inconsistency	no serious indirectness	no serious imprecision	reporting bias[3]	47/218 (21.6%)	55/224 (24.6%)	⊕⊕OO LOW	IMPORTANT
								30.4%		
**Elevation of IOP (follow-up 12 months)**										
4	randomised trials	serious[1]	no serious inconsistency	no serious indirectness	no serious imprecision	reporting bias[3]	101/264 (38.3%)	20/257 (7.8%)	⊕⊕OO LOW	IMPORTANT
								3.3%		
**Adverse events: Cataract (follow-up 12 months)**										
3	randomised trials	serious[1]	no serious inconsistency	no serious indirectness	no serious imprecision	reporting bias[3]	44/254 (17.3%)	15/247 (6.1%)	⊕⊕OO LOW	IMPORTANT
								7.1%		

[1] In one of the studies, the outcome assessors were not blinded for any outcomes

[2] Precision would be rated down by one level due to the small sample size (n<400)

[3] Study size (small studies) or industry sponsorship or other conflicts of interest

[4] Consider the forest plot with included studies where the confidence intervals do not overlap. The p value for heterogeneity is less than 0.05, and I^2 is more that 50%.
